# Characteristics of lung resistance and elastance associated with tracheal stenosis and intrapulmonary airway narrowing in ex vivo sheep lungs

**DOI:** 10.1186/s12931-024-02959-z

**Published:** 2024-09-09

**Authors:** Yuto Yasuda, Geoffrey N. Maksym, Lu Wang, Pasquale Chitano, Chun Y. Seow

**Affiliations:** 1 Centre for Heart Lung Innovation, St. Paul’s Hospital, Providence Health Care, University of British Columbia, 1081 Burrard Street, Rm 166, Vancouver, BC V6Z 1Y6 Canada; 2 School of Biomedical Engineering, Dalhousie University, 6299 South St, Halifax, NS B3H 4R2 Canada; 3 Department of Pathology and Laboratory Medicine, University of British Columbia, Vancouver, BC V6Z 1Y6 Canada

**Keywords:** Central airway obstruction, Peripheral airway narrowing, Lung mechanics, Ventilation frequency, Transpulmonary pressure, Airway smooth muscle

## Abstract

**Background:**

Understanding the characteristics of pulmonary resistance and elastance in relation to the location of airway narrowing, e.g., tracheal stenosis vs. intrapulmonary airway obstruction, will help us understand lung function characteristics and mechanisms related to different airway diseases.

**Methods:**

In this study, we used ex vivo sheep lungs as a model to measure lung resistance and elastance across a range of transpulmonary pressures (5–30 cmH_2_O) and ventilation frequencies (0.125–2 Hz). We established two tracheal stenosis models by inserting plastic tubes into the tracheas, representing mild (71.8% lumen area reduction) and severe (92.1%) obstructions. For intrapulmonary airway obstruction, we induced airway narrowing by challenging the lung with acetylcholine (ACh).

**Results:**

We found a pattern change in the lung resistance and apparent lung elastance as functions of ventilation frequency that depended on the transpulmonary pressure (or lung volume). At a transpulmonary pressure of 10 cmH_2_O, lung resistance increased with ventilation frequency in severe tracheal stenosis, whereas in ACh-induced airway narrowing the opposite occurred. Furthermore, apparent lung elastance at 10 cmH_2_O decreased with increasing ventilation frequency in severe tracheal stenosis whereas in ACh-induced airway narrowing the opposite occurred. Flow-volume analysis revealed that the flow amplitude was much sensitive to ventilation frequency in tracheal stenosis than it was in ACh induced airway constriction.

**Conclusions:**

Results from this study suggest that lung resistance and apparent elastance measured at 10 cmH_2_O over the frequency range of 0.125-2 Hz can differentiate tracheal stenosis vs. intrapulmonary airway narrowing in ex vivo sheep lungs.

## Introduction

Disturbance of airflow can be caused by airway narrowing, which leads to dyspnea or hypoxemia. The airway is anatomically subdivided into the upper and lower airways. The upper airways refer to the nose, nasal cavity, pharynx, and larynx, while the lower airways are composed of trachea, bronchi, and bronchioles [1]. The trachea is both extrathoracic and intrathoracic, whereas the rest of the airway tree is intrathoracic. Upper airway narrowing is usually observed in mechanical obstructions of the airway, which is easily confirmed by physical examination and imaging analysis. However, observation of the lower airway narrowing is usually more difficult. The lower airways can be divided into central and peripheral airways. Tracheal and mainstem bronchial stenoses are examples of central airway obstructions, whereas the constriction of airways seen in airway diseases such as asthma is an example of intrapulmonary airway obstruction, which mainly involves narrowing of small airways [2]. Both types of airway obstructions can cause dyspnea, but the treatments for the dyspnea associated with each type of airway obstruction can be very different.

Understanding the mechanism of how obstructions can affect lung resistance and elastance at different locations of the airway tree, can lead to better design of strategies in improving lung function in different type of lung diseases.

Previous studies have identified several features of dynamic functional properties of the lung associated with diseases affecting upper and lower airways. In differentiating between laryngotracheal and small airway stenoses, expiratory disproportion index (EDI) from spirometry, i.e., the ratio of forced expiratory volume in 1 s (FEV1) to peak expiratory flow rate (PEFR), has been shown to be useful [3]. However, FEV1 measurement is dependent on patient cooperation, meaning that sufficient effort from the patients together with their ability to follow instructions are crucial, and therefore it is not suitable for children or unconscious patients. For tracheobronchomalacia, pulmonary function testing has been shown to be not very effective as a diagnostic tool [4]. A study of airflow dynamics revealed that in tracheal stenosis, the pressure-drop over the segment of stenosis changes drastically between 70 and 80% obstruction of the airway lumen [5]. This is perhaps why patients with tracheal stenosis often report a sudden onset of breathing impairment. The appearance of breathing difficulty in these patients is observed to be associated with a loss of ~ 75% or more of the tracheal lumen [6], while a computed tomography (CT) diagnosis of tracheal stenosis less than 75% blockage is often not associated with a change in the lung function. This is a critical range, because, as a small incremental increase in the blockage above 75% stenosis can result in a drastic change in the lung function. CT diagnosis of airway stenosis therefore can lead to poor reliability in predicting lung function [6]. Furthermore, CT is not capable of detecting small airway obstruction (< 2 mm in diameter) [7] due to its low spatial resolution.

The goal of this study is to further our understanding on how lung function is affected by obstruction of airways at different locations of the airway tree, and to develop a set of criteria for functional measurements of airway obstruction based on the changes in lung resistance and elastance, as well as the flow-volume characteristics, over a range of ventilation frequencies and transpulmonary pressures. The ventilation frequencies used in the present study is much lower than those used in oscillometry (OSC) or forced-oscillation technique (FOT) studies. Findings from this study therefore filled some gaps in our understanding on the changes in the dynamic functional properties of the lung in the low frequency range. The present results also revealed intricate dependence of lung resistance and elastance on ventilation frequency and transpulmonary pressure. Measurements of frequency-dependent flow-volume characteristics in the present study (which is independent of transpulmonary pressure) could also help in distinguishing tracheal stenosis from small airway narrowing due to bronchoconstriction.

## Methods

### Sheep lung preparation

A local halal abattoir kindly provided sheep lungs which were obtained from ~ 8-month-old adolescent sheep. Information regarding the sex of the animals was not available. The isolated whole lungs were put into modified Krebs solution at 4 °C, which contained 118 mM NaCl, 4 mM KCl, 1.2 mM NaH_2_PO_4_, 22.5 mM NaHCO_3_, 2 mM MgSO_4_, 2 mM CaCl_2_, and 2 g/L dextrose. The freshly excised lungs were used for experiments on the same day. Before starting the experiment, the lung was completely inflated by positive pressure air (~ 40 cmH_2_O) to resolve atelectasis. If there were small leaks, tissue glue (sometimes with parafilm) was used to repair them. When the leakage was too big, the lung was discarded.

### Plethysmograph settings

A previously custom-built plethysmograph was used [8]. The temperature inside the plethysmograph was continuously monitored and maintained at 36–39 °C and the humidity at 100% during the experiment. The trachea was connected to a tube through the air-tight ventilation chamber so that the opening of the trachea was exposed to atmospheric pressure, with the dorsal side up and the lung was inflated by negative pressure inside the plethysmograph. Airflow was measured by a pneumotachograph connected to the trachea opening, which means that the pneumotachograph was not subjected to common mode errors. Calibration of the pneumotachograph has been described previously [8]. When the lung was completely inflated, the negative pressure was set to 30 cmH_2_O to start the experiment. Lung resistance and elastance were measured at different transpulmonary pressures from 30 down to 5 cmH_2_O, and in that order. We found that if the experiments were to start from low to high transpulmonary pressures, we were not able to eliminate atelectasis at low pressures.

### Experimental procedure

**Fig. 1:**
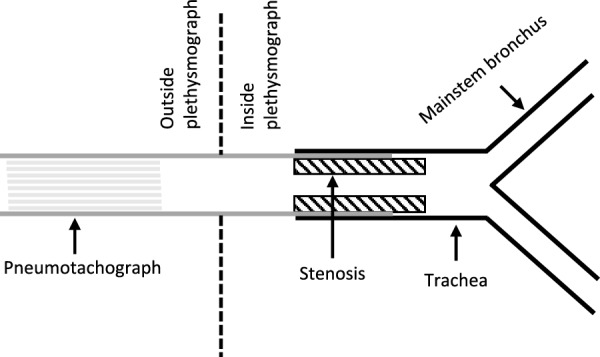
An illustration of a tracheal stenosis model. The black lines outline the trachea and main stem bronchi, the gray lines represent the tube that is part of the pneumotachograph that connects the trachea to the outside of the plethysmograph. The rectangles containing diagonal lines represent a thick-walled polyvinyl tube that creates stenosis inside the trachea

Two tracheal stenosis models were established by using polyvinyl chloride tubes that resulted in the reduction of the tracheal lumen area by 71.8% (mild stenosis model) and 92.1% (severe stenosis model), respectively. Figure 1 illustrates one of the models. The lung resistance (*R*_*L*_) and apparent lung elastance (*E*_*LA*_), described in more detail below, were measured before and after the stenosis was created in the same lungs. Eight lungs were used for each of the two sets of stenosis experiments. *R*_*L*_ and *E*_*LA*_ were measured at 4 different transpulmonary pressures, i.e., 30, 20, 10, and 5 cmH_2_O, and 5 ventilation frequencies, i.e., 0.125, 0.25, 0.5, 1, and 2 Hz. The pressure oscillations inside the plethysmograph were created by a piston whose displacement was driven by a single frequency sinusoid with peak-to-peak volume ventilation set at 0.1 L, superimposed on the mean static transpulmonary pressure. Note that the 0.1 L volume is the amount of air pushed into the plethysmograph by the piston, not the tidal lung volume, which was less than 0.1 L and was variable (frequency dependent). A “resting” period of 1 min was allowed after a pressure change to permit the lung volume to reach a steady state, and the airflow out of the tracheal to reach zero. For measurements at low frequencies (0.125–0.5 Hz), the data acquisition period was set to 80 s, whereas for measurements at higher ventilation frequencies (1 and 2 Hz), the data acquisition period was set to 40 s. However, in data analysis, only 8 cycles of data in the middle of the recorded trace were used to avoid transient changes immediately after a pressure change.

For the model of intrapulmonary airway narrowing, the lung was challenged with nebulized acetylcholine (ACh) (Sigma-Aldrich, Massachusetts). ACh was diluted with modified Krebs solution. Nebulized ACh was delivered to the lung through trachea during tidal ventilation at 10 cmH_2_O of transpulmonary pressure and a sinusoidal ventilation frequency of 0.25 Hz. The concentration of ACh was increased, by doubling the dose from 4 mg/mL and up to 128 mg/mL, until the flow through the trachea was decreased by 30–50%. If 128 mg/mL of ACh was reached and the flow did not reach the targeted range, the lung was not used for the experiment. The lung resistance and elastance were measured before and after ACh challenge in the same lungs, using the same transpulmonary pressures and ventilation frequencies as the stenosis models.

### Calculation of lung resistance and elastance

**Fig. 2:**
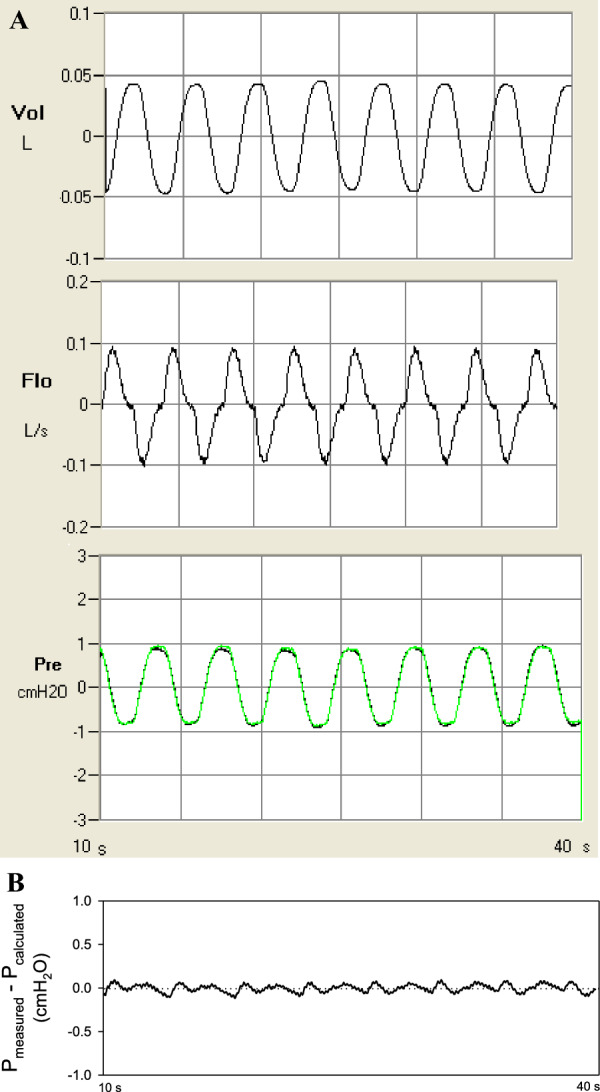
**A** Examples of raw data from experiments fitted with model predictions. Upper panel shows the volume trace integrated from the flow trace (middle). The lower panel shows recorded transpulmonary pressure trace (black) superimposed with calculated pressure (green) based on the volume and flow data from the upper and middle panels. Goodness of fit r^2^ = 0.999. **B** Difference between recorded and calculated transpulmonary pressures

The calculation of lung resistance and elastance at each frequency of oscillation was based on an established linear single-compartment time domain model fit to the pressure and flow signals as described by Bates [9]. Briefly, the time domain model for the transpulmonary pressure is as follows:

1 $${P}_{L}={E}_{LA}V+{R}_{L}\dot{V}+{P}_{0}$$

where *P*_*L*_ is the transpulmonary pressure, *E*_*LA*_ is the apparent lung elastance, *R*_*L*_ is lung resistance, ${\dot{\text{V}}}$ is the airflow,* V* is the ventilation volume calculated from integrating the flow signal, and *P*_*0*_ is an offset pressure which sets *P*_*L*_ = mean transpulmonary pressure when both *V* and ${\dot{\text{V}}}$ are zero. As illustrated in Fig. 2A, the volume trace (upper panel) was obtained by integrating the flow trace (middle panel). The recorded pressure trace (raw data, lower panel) was plotted in black, while the calculated pressure trace (from Eq. 1) was plotted in green. The goodness of fit (r^2^) between recorded and calculated pressure traces for data obtained in this study was all greater than 0.99. In Fig. 2B, the difference between the recorded and calculated pressure traces is plotted. No systematic deviation between the two traces was observed. Since single frequency perturbation was applied, only two parameters can be identified from the modelling, *R*_*L*_ and *E*_*LA*_. At low frequencies, the two compartment model is valid with *E*_*LA*_ being nearly entirely elastic, but as we measured at multiple frequencies we were able to identify inertive effects contributing to *E*_*LA*_ with increasing frequency [10]. Since elastance measured (using Eq. 1) at high frequency contains a significant component of inertial effect, we call the measured elastance “apparent” elastance (*E*_*LA*_). The single compartment description of *R*_*L*_ and *E*_*LA*_ for our single frequency approach follows the approach of Kaczka et al. [11, 12] and Lutchen et al. [13] for simplicity as the *R*_*L*_ and *E*_*LA*_ can be easily obtained by a least-square best-fit between the pressure values predicted by Eq. 1 at each frequency and the recorded pressure data from experiments without any user defined assumptions and bias, as described by Bates [9]. The shunt effect of alveolar gas compressibility on the proximal (airway) compartment was ignored because of the relatively low ventilation frequencies used in the measurements.

*R*_*L*_ and *E*_*LA*_ were calculated as follows for *N* number of digitized data points:

2 $${R}_{L}=\frac{\sum_{i=1}^{N}{V}_{i}^{2}\sum_{i=1}^{N}{\dot{V}}_{i}{P}_{i} - \sum_{i=1}^{N}{V}_{i}{\dot{V}}_{i}\sum_{i=1}^{N}{V}_{i}{P}_{i}}{\sum_{i=1}^{N}{V}_{i}^{2}\sum_{i=1}^{N}{\dot{V}}_{i}^{2} -{ \left(\sum_{i=1}^{N}{V}_{i}{\dot{V}}_{i}\right)}^{2}}$$

3 $${E}_{LA}=\frac{\sum_{i=1}^{N}{V}_{i}{P}_{i}-{R}_{L}\sum_{i=1}^{N}{V}_{i}{\dot{V}}_{i}}{\sum_{i=1}^{N}{V}_{i}^{2}}$$

### Statistical analysis

Two-way repeated measures ANOVA (factors: frequency and control/stenosis) and Holm-Sidak all pairwise multiple comparison post-hoc tests were used for statistical analyses. Standard errors of means were used to plot the graphs. Statistically significant difference was set at p < 0.05. All analysis was performed by using SigmaPlot 14.0.

## Results

In control measurements at transpulmonary pressures > 5 cmH_2_O, *R*_*L*_ and *E*_*LA*_ showed consistent dependence on respiratory frequency across the three sets of experiments. *R*_*L*_ decreased with increasing frequency, while *E*_*LA*_ increased with increasing frequency at 10, 20, and 30 cmH_2_O transpulmonary pressures, with statistical significance reached in 14 out of the 18 subgroups of data. These opposite responses of *R*_*L*_ and *E*_*LA*_ were not detected at a low lung volume (transpulmonary pressure of 5 cmH_2_O) due to large standard deviations associated with the data. This could be due to variability associated with airway opening and closing that could occur at low lung volumes, affecting the mechanics. It is possible that small pockets of atelectasis exist at this low lung volume in ex vivo lungs. The results suggest that to detect changes in resistance and elastance, measurements made at low lung volumes may not yield the best resolution with our present experiment setup and protocol.

### Tracheal stenosis models

Table 1 lists the dimensions of the plastic tubes used to create tracheal stenoses. Because the only difference between mild and severe stenosis models is the diameter (the tube length is the same), one could calculate the resistance difference due to the stenoses per se. According to Poiseuille’s Law for laminar flow, resistance through a tube for a particular fluid is defined as 8η*l*/πr^4^, where η is the fluid viscosity, *l* is the tube length, and r is the inner diameter of the tube. Since η and *l* are the same for both stenosis models, the only difference is r. Without stenosis, the resistance is 8η*l*/π(5.7)^4^; for mild stenosis, the resistance is 8η*l*/π(3.025)^4^; and for severe stenosis, the resistance is 8η*l*/π(1.6)^4^. From the above, one can calculate that there is a 12.6-fold increase in resistance relative to control for mild stenosis model, and for severe stenosis, there is a 161.07-fold increase in resistance relative to control. These numbers do not match those measured from the whole lungs (as expected, as shown below), because the resistance stemming from the stenosis per se is only a part of the complex lung resistance.

**Table 1:** Tube dimensions for control, mild and severe stenosis models

	Inner Diameter (mm)	Length (mm)	Lumen Area (mm^2^)
Control	11.4	5.0	102.07
Mild Stenosis	6.05	3.5	28.75
Severe Stenosis	3.2	3.5	8.04

**Fig. 3:**
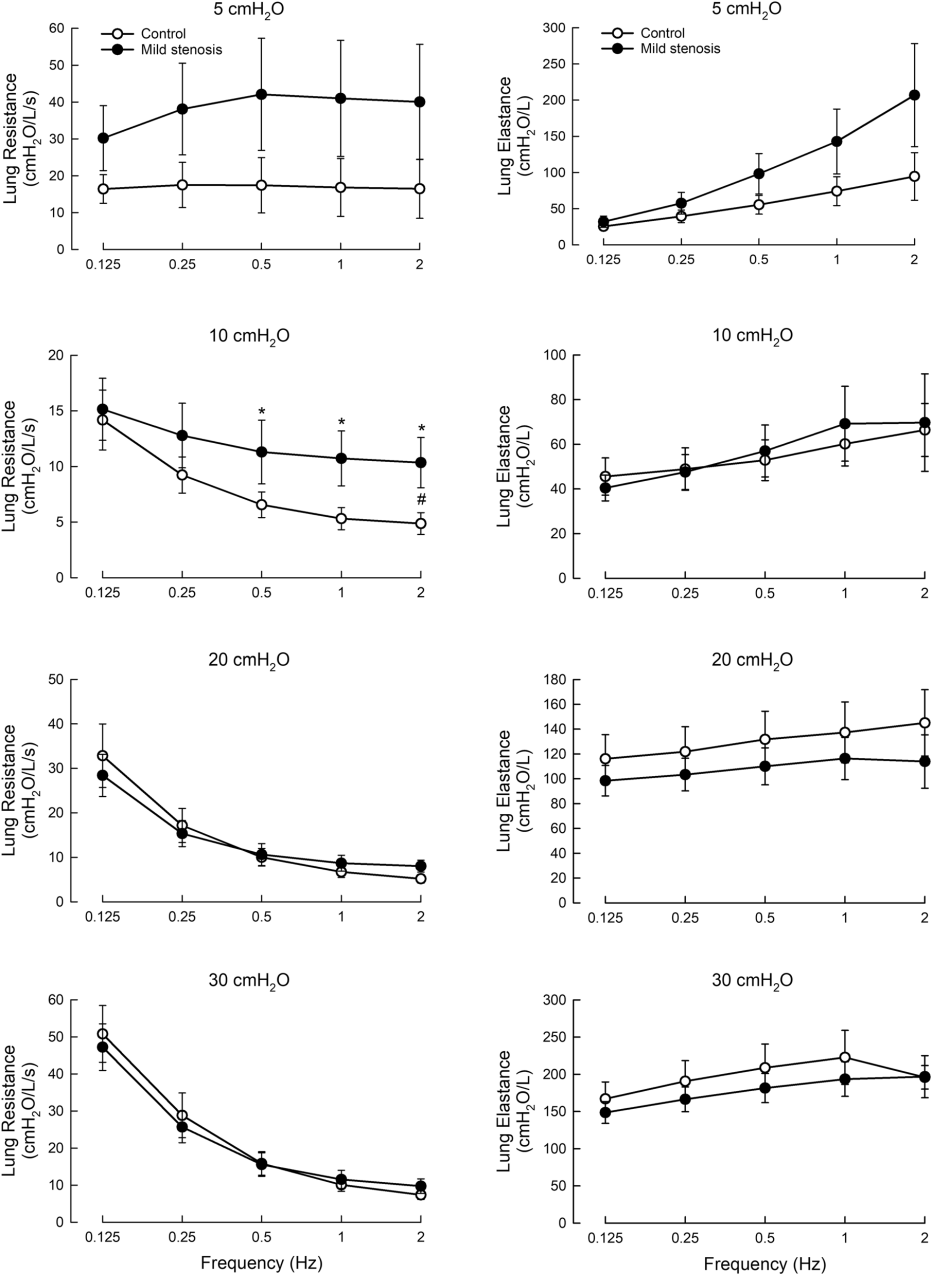
*R*_*L*_ and *E*_*L*_ in mild tracheal stenosis model at varied ventilation frequencies and transpulmonary pressures. ANOVA as described in Methods were conducted. Open symbols, control; solid symbols, stenosis model. N = 8. *, difference between corresponding values (same frequency) in control and test, p < 0.05; #, difference between control and test groups, p < 0.05

In the mild tracheal stenosis model (71.8% blockage, Fig. 3, left panels), we found a statistically significant increase in *R*_*L*_ when compared with the control (0% blockage) at the transpulmonary pressure of 10 cmH_2_O. For *E*_*LA*_ (Fig. 3, right panels), no significant difference was found between the test and control groups.

**Fig. 4:**
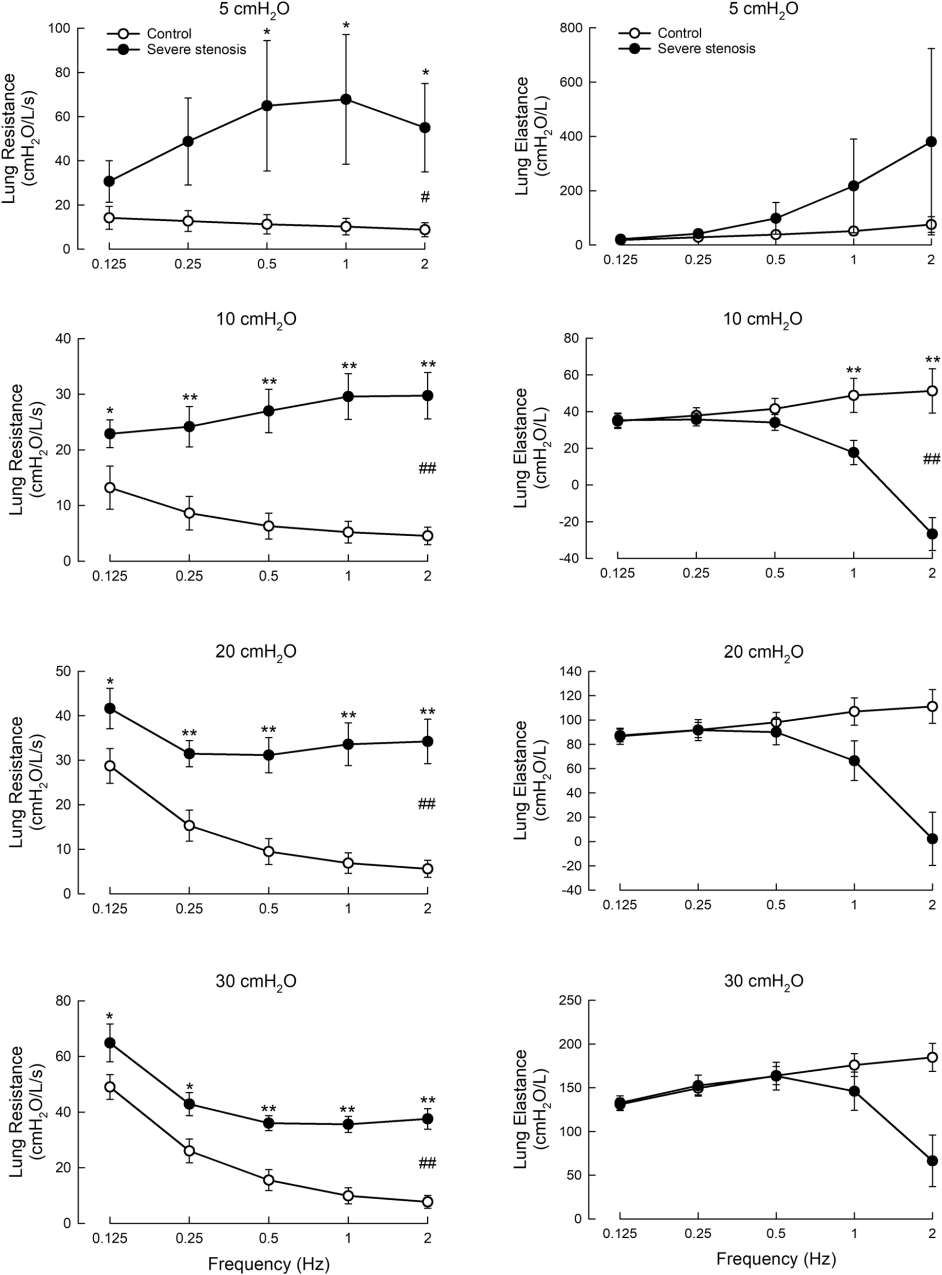
*R*_*L*_ and *E*_*L*_ in severe tracheal stenosis model at varied ventilation frequencies and transpulmonary pressures. ANOVA as described in Methods were conducted. Open symbols, control; solid symbols, stenosis model. N = 8. *, difference between corresponding values (same frequency) in control and test, p < 0.05; **, p < 0.01; #, difference between control and test groups, p < 0.05, ##, p < 0.01

In the model of severe tracheal stenosis (92.1% blockage, Fig. 4), the increase in *R*_*L*_, when compared to the control, was greater than in mild stenosis, being significant at all transpulmonary pressures. Interestingly, *R*_*L*_ at 5 and 10 cmH_2_O increased with increasing ventilation frequency (solid symbols), whereas at higher pressures (20 and 30 cmH_2_O) it decreased with increasing frequency, reaching a plateau at a frequency of 0.5 Hz or greater.

In the severe tracheal stenosis model, *E*_*LA*_ exhibited a different pattern of frequency dependence compared with the controls (Fig. 4, right panels). While in the control, *E*_*LA*_ increased monotonically with ventilation frequency, in severe stenosis it showed a biphasic response, being not different from the control at low frequencies and decreasing significantly at higher frequencies. This response was observed at transpulmonary pressures of 10, 20, and 30 cmH_2_O. At 5 cmH_2_O no statistical difference was found between control and severe stenosis. The results showed that to differentiate *E*_*LA*_ of the severe stenosis group from the control, measurements have to be done at a transpulmonary pressure of 10 cmH_2_O or greater, and at ventilation frequencies of 1 Hz or higher.

### The model of intrapulmonary airway narrowing

**Fig. 5:**
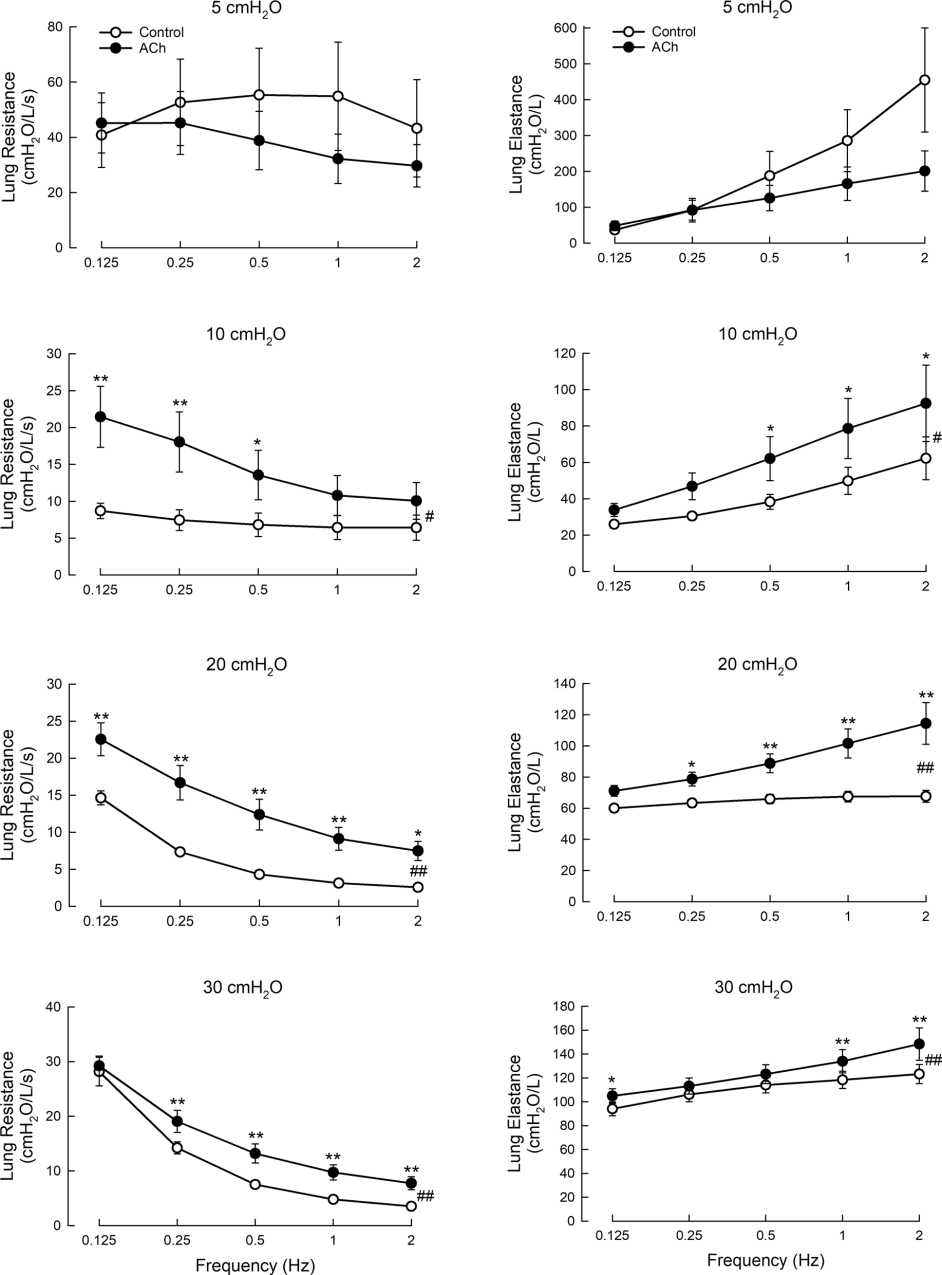
*R*_*L*_ and *E*_*L*_ in the acetylcholine-induced airway narrowing model at varied ventilation frequencies and transpulmonary pressures. ANOVA as described in Methods were conducted. Open symbols, control; solid symbols, airway narrowing model. N = 9. *, difference between corresponding values (same frequency) in control and test, p < 0.05; **, p < 0.01; #, difference between control and test groups, p < 0.05, ##, p < 0.01

While no statistical difference was found at 5 cmH_2_O, *R*_*L*_ in ACh-challenged lungs at 10–30 cmH_2_O transpulmonary pressures was observed to be significantly greater than the control values (Fig. 5, left panels). The pattern of frequency-dependent change in *R*_*L*_, however, is the same in both control and test groups, i.e., the resistance decreases with increasing frequency and the test group data are simply shifted upward.

Also, for *E*_*LA*_, while no statistical difference was found in ACh challenged lungs compared with controls at 5 cmH_2_O, *E*_*LA*_ measurements were significantly greater than their respective controls at 10, 20 and 30 cmH_2_O (Fig. 5, right panels). The elastances in these groups showed a monotonic increase with ventilation frequency, a pattern that is very different from that observed in severe tracheal stenosis (Fig. 4, right panels).

### Changes in the flow-volume characteristics

**Fig. 6:**
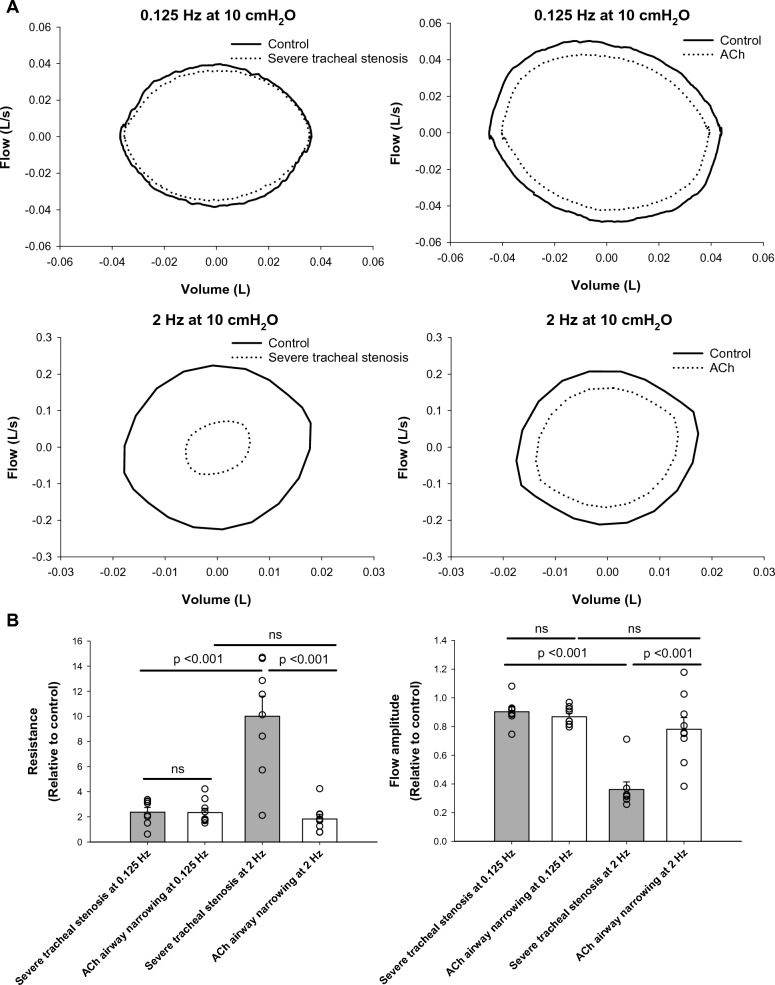
**A** Examples of flow-volume curves obtained at a transpulmonary pressure of 10 cmH_2_O and two ventilation frequencies (0.125 and 2 Hz) for the severe tracheal stenosis model and ACh stimulated airway narrowing. The curves were obtained from averaging individual flow-volume loops from all experiments (n = 8 for severe tracheal stenosis; n = 9 for ACh-induced airway narrowing). **B** Lung resistance and flow amplitude (peak-to-peak) measured at 0.125 and 2 Hz of ventilation frequency and 10 cmH_2_O of transpulmonary pressure in severe tracheal stenosis and ACh induced airway narrowing. Means and standard errors are plotted along with individual data points from all experiments (n = 8 for severe tracheal stenosis; n = 9 for ACh-induced airway narrowing)

Figure 6A shows an example of flow-volume curves obtained under different conditions. Averaged curves are plotted, representing the flow-volume loops obtained at a transpulmonary pressure of 10 cmH_2_O and ventilation frequency of 2 Hz in severe tracheal stenosis and airway narrowing by ACh stimulation. Figure 6B shows changes in lung resistance (left panel) and (peak-to-peak) flow amplitude (right panel) with different ventilation frequencies at a transpulmonary pressure of 10 cmH_2_O. It is clear from Fig. 6B that the resistance (relative to control) increased with frequency in tracheal stenosis, while the resistance decreased (although not significantly) with frequency in airway narrowing induced by ACh. From Fig. 6B it is also clear that the flow amplitude was highly sensitive to ventilation frequency in tracheal stenosis, whereas in the scenario of airway narrowing by ACh, the flow amplitude was insensitive to ventilation frequency.

## Discussion

The major findings of the present study are that the frequency-dependent lung resistance and elastance behave differently when airway narrowing occurred at different locations of the airway tree (tracheal stenosis vs. ACh stimulated airway constriction); the flow-volume characteristics also exhibited different sensitivity to ventilation frequency depending on the location of airway obstruction. These differences allow us to gain insight into the mechanisms underlying changes in lung function due to airway obstruction associated with different diseases.

The incidence of tracheal and central airway stenosis is increasing primarily due to the increase in lung cancer cases [14]. Non-cancer-related tracheal stenosis, often associated with tracheal intubation, tracheotomy and tracheomalacia, has also contributed to the trend [14, 15]. Medical and surgical solutions are required to treat tracheal or central airway stenosis, such as stent placement [16]. As many as 4 out of 5 patients with laryngotracheal stenosis have been routinely misdiagnosed [17], and incorrectly treated for many years as bronchopulmonary diseases such as asthma [18, 19]. The diagnostic delay will obviously increase the risk of treatment failure and missed window for intervention in tracheal or central airway cancers [20]. Although most of the methodologies described in the present study cannot be directly adapted in the development of a practical diagnostic device for differentiating locations of airway obstruction, mainly because transpulmonary pressure cannot be readily measured in human subjects. But, knowing that there are differences in the dynamic functional properties of the lung associated with different locations of airway obstruction may inspire future studies to develop non-invasive methods for detecting locations of airway obstruction. This goal is made more feasible by our finding that the flow-volume characteristics, which can be determined without the measurement of transpulmonary pressure, have different sensitivity to ventilation frequency depending on the location of airway obstruction.

### Interpretation of our observations

The control data show that *R*_*L*_ was constant with frequency at 5 cmH_2_O of transpulmonary pressure, but exhibited an inverse frequency dependence at higher pressures, likely attributable to the known frequency dependence of lung tissue. The increasing dependence on pressure is likely due to the greater stretch of the tissue at higher pressure, increasing its contribution to *R*_*L*_. Changes in airway heterogeneity could also be a factor underlying the changes in *R*_*L*_ with frequency and pressure [21]. The increase in *E*_*LA*_ with pressure across all frequencies is similarly likely due to the increased stretch of the lung tissue and thus greater stiffness due to the stress–strain nonlinearity of lung tissue [22] (Figs. 4 and 5).

In all control groups (open circles in Figs. 3–5), large variance was found in the measurements of *R*_*L*_ and *E*_*LA*_ at 5 cmH_2_O. At this transpulmonary pressure *R*_*L*_ was also found to be much higher than that reported by others investigating lung mechanics in difference species, including humans [23] and dogs [24]. Our previous studies using the same isolated sheep lungs [7, 25] also found elevated *R*_*L*_ at FRC. This may suggest sheep lungs possess high intrinsic *R*_*L*_. Our unpublished observation reveals that spontaneous increase in *R*_*L*_ over time occurs in isolated sheep lungs, which can be abolished by a simulated deep inspiration. This suggests that there is spontaneous airway smooth muscle tone that develops over time. We do not know whether this is a general phenomenon in sheep lungs, or it is a phenomenon of the particular population of sheep we used for our experiments. The elevated *R*_*L*_ could also be due to the particular protocols used in our experiments where the lungs were ventilated with small tidal volume for a long period of time (30 min) without any deep inspiration. The developed smooth muscle tone can explain the elevated *R*_*L*_, but the lack of frequency-dependent decrease in *R*_*L*_ at 5 cmH_2_O found in the present study (in contrast to that observed by Brusasco et al. [24]) is more difficult to explain. Bates et al. [26] found that lung tissue resistance is low at low lung volumes (or low transpulmonary pressures), and increases linearly with lung volume. Since tissue resistance decreases with ventilation frequency, our finding of *R*_*L*_ being frequency independent at 5 cmH_2_O suggests that there is no tissue resistance in sheep lungs at this low lung volume, or the portion of the tissue resistance within *R*_*L*_ is very small. Maybe the large variation in the measured *R*_*L*_ somehow obscured the small changes in *R*_*L*_ with frequency. The source of the large standard deviation could be due to random closing and reopening of airways at low lung volume, perhaps due to liquid-bridging, i.e., airway closure due to the formation of occluding liquid bridges across the airway lumen [27], which tends to occur more often in small airways and it effectively reduces the ventilable lung volume. In our measurements, besides large variability, values of *E*_*LA*_ at 5 cmH_2_O were found to be much higher than those measured at higher transpulmonary pressures, especially at high ventilation frequencies. This could also be due to closure of small airways at low lung volume, which effectively reduced the ventilable volume of the lung. A smaller lung volume would make the lung appear stiffer, just like a shorter spring would appear stiffer than a longer spring when one stretches them by the same amount.

*R*_*L*_ and *E*_*LA*_ measured in this study were based on a simplified linear single-compartment model of the lung (Eqs. 1–3) that ignored the influence of inertance [9]. This is commonly done for low ventilation frequencies (near tidal frequency) in adult humans and relatively large animals such as sheep with little error in healthy lungs [28], however, effects of inertance are well understood to manifest with increasing frequency and faster flows, and will begin to largely affect the interpreted elastance of the simplified single-compartment model we used in this study. The inertance effect causes the apparent elastance, *E*_*LA*_, to decrease with increasing frequency and it explains the behavior seen in Fig. 4 (beginning at 0.5 Hz and up) as *E*_*LA*_ decreased with increasing ventilation frequency.

If we ignore the measurements made at the low lung volume corresponding to a low transpulmonary pressure of 5 cmH_2_O where the standard deviations are large and no clear frequency dependence is found, the general observation in all control groups (non-stenotic, open circles in Figs. 3–5) is that *R*_*L*_ decreases and *E*_*LA*_ increases with increasing ventilation frequency. This is consistent with the observations made in previous studies [29–31]. As observed in sheep [32], for a wide range of pressures, the frequency dependence of *R*_*L*_ increased with the pressure in healthy lungs, which is consistent with our observations here. At low ventilation frequencies, *R*_*L*_ is dominated by tissue resistance, but this component of resistance diminishes as frequency increases and at frequencies greater than 2–4 Hz, the measured lung resistance is almost entirely made of airway resistance, which is relatively frequency independent [10, 33]. Indeed, Kaczka et al. [11] utilized this distinct difference in frequency response to partition airway and tissue resistances in their measurement of lung resistance.

Our inability to reliably measure lung resistance and elastance at the low pressures of 5 cmH_2_O is obviously a limitation of the study, because at that pressure the lung volume is approximately at the functional residual capacity. Measurements of lung function at pressures higher than the natural end expiratory pressure in human subjects requires externally applied positive pressure which raises the end expiratory pressure.

The increase in *E*_*LA*_ with increasing ventilation frequency in the low frequency range (0.125–0.25 Hz) is consistent with the known frequency dependence of compliance, the inverse of elastance, which decreases with frequency. At higher frequencies (< 0.5 Hz) the inertial effects gave rise to the decrease in the modeled *E*_*LA*_ and it became negative at 2 Hz. Severe narrowing in the trachea will increase oscillatory flow and accelerations particularly at higher frequencies, giving rise to negative *E*_*LA*_ as observed in human subjects above a ventilation frequency of 2 Hz [11].

In ACh challenged lungs (Fig. 5) where narrowing of the intrapulmonary airways occurred, the general behavior of the frequency-dependent change in *R*_*L*_ and *E*_*LA*_ was the same as the control, except that both the resistance and elastance in the challenged lungs were elevated compared with the control. The increase in *R*_*L*_ due to ACh exposure is expected due to airway constriction and maybe increase in airway heterogeneity as well [21]. The upward shift of the lung resistance vs. frequency has also been observed in asthma [12, 13]. Stiffer airways due to airway smooth muscle contraction might contribute to the increase lung elastance, however, atelectasis created by airway closure is likely the major source for the increase in lung elastance. Our recent observation in the same ex vivo lung preparation also yields the same results regarding changes in resistance and elastance due to ACh stimulation [25].

In our model of mild tracheal stenosis (Fig. 3), the only statistically significant difference we observed is the *R*_*L*_ measured at 10 cmH_2_O under the stenotic and control conditions. Therefore, there was a narrow window to detect an increase in *R*_*L*_ in mild tracheal stenosis, i.e., at a transpulmonary pressure around 10 cmH_2_O and at a ventilation frequency greater than that of natural resting tidal breathing frequency of 0.25 Hz. This is consistent with the finding that the onset of breathing difficulty in patients with tracheal stenosis is often not detected when a loss of the airway lumen is less than 75% [6]. We do not know why at this particular transpulmonary pressure (10 cmH_2_O) the measurement is the most sensitive, but considering that lung resistance is made of airway resistance and tissue resistance, it may be that the increase in airway resistance from the stenosis at 10 cmH_2_O is of greater influence than the increase in lung tissue resistance arising from the stretch of the lung tissue [22]. For *E*_*LA*_ (Fig. 3, right panels), no significant difference was found between the test and control groups. The results suggest that it would be difficult to detect a change in elastance in patients having tracheal stenosis with blockage that is less than 75%.

In our model of severe tracheal stenosis (Fig. 4), the difference in *R*_*L*_ and *E*_*LA*_ between the control and stenosis groups are obvious. For the measurements made at 5 and 10 cmH_2_O, *R*_*L*_ (Fig. 4, left panels) for the stenotic group (solid symbols) increased with ventilation frequency, opposite to the control. This could be due to turbulence at the stenosis insert which can occur due to the higher flows leading to increases in resistance in a frequency dependence manner [34]. At higher pressures (20 and 30 cmH_2_O), *R*_*L*_ for the stenotic group (solid symbols) again exhibits the decreasing frequency dependence likely due to the increased stretch of the lung tissue (Fig. 4, left panels). This behavior is expected from tissue resistance dominating the airway resistances with the plateau values of the lung resistance observed in the stenotic group, therefore represents an elevated airway resistance.

For the severe stenosis model, *E*_*LA*_, besides that measured at 5 cmH_2_O where no statistical difference is found, showed a biphasic behavior (Fig. 4, right panels). Initially it behaved like the control, i.e., increases with ventilation frequency up to 0.5 Hz, then it decreased at higher frequencies (Fig. 4, right panels). As we described below, our interpretation of *E*_*LA*_ neglects the contribution of inertance. At higher frequencies, Turner et al. have shown that while the resistance is not affected by the omission of inertance, errors (*ΔE*) in the estimate of elastance increase as *ΔE* = -ω2*I*, where ω = 2π*f* (ventilation frequency) and *I* is the inertance [35]. For small animals (with small tracheas), Lanteri et al. found that the contribution of inertance starts to become significant at frequencies greater than 0.3–0.5 Hz [36]. Our observation in the stenotic model showed that the frequency dependent behavior is similar to that observed in small animals, suggesting that this is a general behavior when the diameter of the tracheas is small. *E*_*LA*_ measured at higher frequencies in the stenotic model shows a different frequency dependence arising from inertial effects and is not constant, but grows with -ω^2^*I*, (further described below) as observed in *E*_*LA*_ decreasing when the ventilation frequency exceeded 0.5 Hz (Fig. 4, right panels). The biphasic frequency-dependent behavior of *E*_*LA*_ has also been observed in human subjects with no central airway stenosis. However, the frequency at which *E*_*LA*_ starts to decline is much higher (> 2Hz) [11, 12]. Tracheal stenosis, as observed in the present study, shifted the curve left-ward, i.e., *E*_*LA*_ started to decline much earlier, when the frequency was greater than 0.5 Hz (Fig. 4, right panels).

**Fig. 7:**
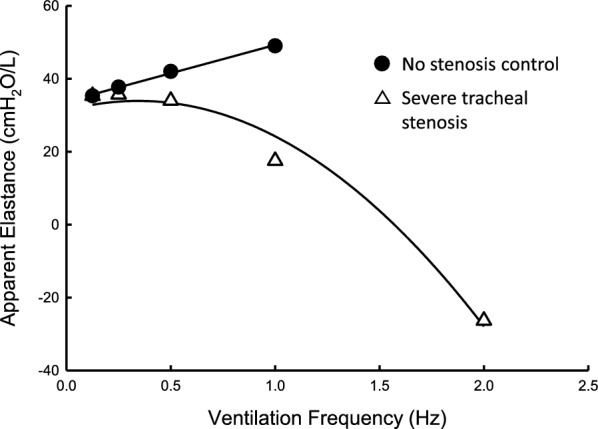
Analysis of *E*_*LA*_ as functions of ventilation frequency, using *E*_*LA*_ from Fig. 4 (10 cmH_2_O). Under control conditions (no stenosis) the *E*_*LA*_ data (solid circles) were fitted with a linear function *E*_0_ + *E*_*1*_·*f* to obtain *E*_0_ = 33.7 (cmH_2_O/Liter), *E*_*1*_ = 15.5 [(cmH_2_O/Liter)/Hz] the coefficient of the linear dependence of *E*_*LA*_ on frequency, and *f* is the ventilation frequency. Note that when inertial effects were apparent visible as a descending curve with frequency, A quadratic fit is more appropriate as for the case of severe tracheal stenosis. Using the *E* value from the linear fit of control data, the *E*_*LA*_ data for the case of severe tracheal stenosis (open triangles) are fitted to *E*_0_ + *E*·*f* + *I*·*f* ^2^ to obtain *I* = − 22.5 [(cmH_2_O/Liter)/Hz^2^]

Figure 7 further illustrates the point that in severe tracheal stenosis the influence of inertance in the mechanics is substantial. Under control conditions where there is no stenosis (solid circles), the inertance can be neglected and the elastance is a linear function of ventilation frequency, at least in the low frequency range. In severe tracheal stenosis, *E*_*LA*_ (open triangles) is no longer a linear function of ventilation frequency. In fact, when inertance, *I*, is included in Eq. (1), giving the standard equation of motion for the respiratory system:

$${P}_{L}=EV+{R}_{L}\dot{V}+I\ddot{V}+{P}_{0}$$

where, *E* is the elastance, $\ddot{V}$ is the acceleration of *V*, and the double dot indicates the second derivative, it can be seen that $I$ is the coefficient of the 2nd derivative of volume. Then for sinusoidal waveforms or any waveform via Fourier decomposition where the double derivative introduces the minus sign, then one can solve for the elastance using least squares from Eq. 1 over frequency by fitting the equation

$${E}_{LA}=E-I{\omega }^{2}$$

where volume is now a function of the radial frequency $\omega$, ($\omega =2\pi f),$ and *E* is the elastic constant. The curve fit is shown in Fig. 7. This shows that the decrease in *E*_*LA*_ seen at high frequencies can be accounted for when inertance is considered.

Miller and Hyatt [37] were able to distinguish variable obstructing lesions of the trachea and larynx based on the difference in the inspiratory and expiratory limbs of the flow-volume loops, but for fixed obstruction of trachea, no difference was found in the inspiratory and expiratory limbs. This is consistent with our findings in fixed tracheal stenosis (Fig. 6). For ACh-induced airway narrowing (an example of variable obstruction), we were not able to quantify such differences in our data (Fig. 6), likely due to the small amplitude of ventilation volume used in our experiments where expiratory flow limitation was not reached. However, we found a distinct difference in the peak-to-peak flow amplitude in terms of its sensitivity to ventilation frequency, and when combined with the *R*_*L*_ measurement, we could differentiate tracheal stenosis from general airway narrowing due to ACh challenge (Fig. 6B).

### Potential applications

Because transpulmonary pressure cannot be easily measured in human patients, indirect measures such as the use of esophageal pressure as a surrogate for transpulmonary pressure is often necessary. This has made the application of the methodology developed in the present study as a non-invasive diagnostic tool impossible. To avoid the difficulty of transpulmonary pressure measurement, Lutchen et al. [38] used a partial-body plethysmograph to non-invasively measure transfer impedance in healthy subjects and patients with lung diseases. They were able to distinguish several features of transfer impedance in different experimental groups. Findings from the present study could help in the interpretation of results from partial or full body plethysmograph measurements, especially if the experiments are designed to detect the location of airway obstruction. Our finding that the peak airflows are different in tracheal stenosis and airway constriction, especially in their dependence on ventilation frequency (Fig. 6). This method does not require any knowledge of transpulmonary pressure, and thus suggests a potential non-invasive method for detecting locations of airway obstruction, although more studies are needed to validate the applicability, reliability, and accuracy of our methods in the diagnosis.

## Conclusions

Tracheal stenosis and intrapulmonary airway narrowing can be differentiated in the pattern of ventilation-frequency-dependent changes of *R*_*L*_ and *E*_*LA*_. The different patterns can be observed most clearly at a transpulmonary pressure of 10 cmH_2_O and a ventilation frequency range of 0.125 to 2 Hz. The difference in the frequency-dependent flow-volume characteristics also provides a potential indicator for airway obstruction at different locations of the airway tree.

## Data Availability

The datasets generated and/or analyzed during the current study are available from the corresponding author on reasonable request.
